# Supplementary material for: Controlling cell shape on hydrogels using lift-off protein patterning
Source: PLoS One. 2018 Jan 3;13(1):e0189901. doi: 10.1371/journal.pone.0189901 (PMC5752030; doi:10.1371/journal.pone.0189901)
Supplement: S1 Table — (DOCX) [file pone.0189901.s001.docx]

| **S1 Table: Polyacrylamide gel formulations used in this study** | | | | | | | | | |
| --- | --- | --- | --- | --- | --- | --- | --- | --- | --- |
| **Stiffness (kPa)** | **%T** | **%C** | **% Acryl.** | **% Bis.** | **Water (µl)** | **Acryl. (µl)** | **Bis. (µl)** | **APS** | **TEMED (µl)** |
|  | **Stock Concentration** | | | | | **0.5 g/ml** | **0.025 g/ml** | **10%** |  |
| 25 | 10.25 | 2.44 | 10 | 0.25 | 694 | 200 | 100 | 5 | 1 |
| 10 | 10.10 | 0.99 | 10 | 0.1 | 754 | 200 | 40 | 5 | 1 |
| 5 | 5.15 | 2.91 | 5 | 0.15 | 834 | 100 | 60 | 5 | 1 |
| %C = Crosslinker | | | | | | | | | |
| %T = Total Monomer (w/v) | | | | | | | | | |
| Acryl. = acrylamide | | | | | | | | | |
| Bis. = bisacrylamide | | | | | | | | | |
