# Supplementary material for: Controlling cell shape on hydrogels using lift-off protein patterning
Source: PLoS One. 2018 Jan 3;13(1):e0189901. doi: 10.1371/journal.pone.0189901 (PMC5752030; doi:10.1371/journal.pone.0189901)
Supplement: S2 Table — (DOCX) [file pone.0189901.s002.docx]

| **S2 Table: Acceptable feature yield results** | | | | | | | |
| --- | --- | --- | --- | --- | --- | --- | --- |
|  | **LO** |  |  |  | **µCP** |  |  |
|  | **Acceptable Number of Features** | **Total Number of Features** | **Acceptable Features (%)** |  | **Acceptable Number of Features** | **Total Number of Features** | **Acceptable Features (%)** |
| **5 kPa** | 231 | 391 | 59.08% |  | 14 | 389 | 3.60% |
| **10 kPa** | 383 | 389 | 98.46% |  | 296 | 410 | 72.20% |
| **25 kPa** | 396 | 418 | 94.74% |  | 160 | 416 | 38.46% |
