# Supplementary material for: Controlling cell shape on hydrogels using lift-off protein patterning
Source: PLoS One. 2018 Jan 3;13(1):e0189901. doi: 10.1371/journal.pone.0189901 (PMC5752030; doi:10.1371/journal.pone.0189901)
Supplement: S1 Text — We also include comparisons between polyacrylamide pore size and gelatin protein molecular size to aid discussion of protein transfer efficiency. (DOCX) [file pone.0189901.s003.docx]

Supporting Information

Controlling cell shape on hydrogels using lift-off protein patterning

Jens Moeller^1,¶^, Aleksandra K. Denisin^1,2,^^¶^, Joo Yong Sim^1^, Robin E. Wilson^1^, Alexandre J.S. Ribeiro^1^, Beth L. Pruitt^1,2,3,4, *^

^1^ Department of Mechanical Engineering, Stanford University, Stanford, California, United States of America

^2^ Department of Bioengineering, Stanford University, Shriram Center, Stanford, California, United States of America

^3^ Stanford Cardiovascular Institute, Stanford University, Stanford, California United States of America

^4^ Department of Molecular and Cellular Physiology, Stanford University School of Medicine, Beckman Center, Stanford, California, United States of America

* Corresponding author

Email: pruitt@stanford.edu (BLP)

^¶^ These authors contributed equally to this work

**I. Lift-off Protocol**

**S1818 photolithography of coverslips**

1. Wash the coverslips sequentially in acetone, isopropyl alcohol, and deionized water until all organic solvents are washed away. Carefully dry the coverslips with nitrogen gas.
2. Place coverslips on a 180°C hotplate for 5 min to complete drying process. When drying is finished store the coverslips again in individual boxes. Set the hotplate to 100°C and place a blank silicon wafer on top.
3. Align a chuck for spinning coverslips in the SU-8 spinner. Align a coverslip on the chuck and turn on the vacuum.
4. Select the following program parameters for the spinning protocol to achieve S1818 layer at 2µm thickness:

5 seconds at 2000 RPM

1 min 30 seconds at 4000 RPM

1. Use the transfer pipette to put ~0.5ml of S1818 onto the coverslip. Run the spin program.
2. When the program is finished, use a lint-free swab, dip in acetone and wipe around the edges of the coverslip to remove photoresist edge bead.
3. Bake at 100°C for 2 minutes. Transfer to individual box when baking is finished for cooling.
4. Expose each coverslip one at a time on the OAI system using a chrome mask of the design. Make sure that you put the coverslip on top of a silicon wafer to decrease back-scattering during exposure. You must achieve 40-50 mJ/cm^2^ at 365 nm intensity for adequate exposure of the S1818. Determine exposure times based on calibration of the light source using a power meter.

1. Develop for 60 seconds in MF319 development solution. Rinse in deionized water, dry fully with nitrogen gas.
2. Verify the structures using a microscope.

**Photoresist lift-off to fabricate protein patterned coverslips**

1. Start with a S1818 photoresist structure on a clean glass slide (see S1818 photolithography protocol)
2. Clean suitable glass beakers (five of 25ml size; one 200ml size) with 2% Hellmanex solution overnight. Wash thoroughly with deionized water afterwards.
3. Fill the 25 ml beakers with N-Methyl-2-pyrrolidone (NMP), water and NMP / water mixture following the following volume ratios:

Beaker 1: 1/3 NMP fraction, 2/3 deionized water fraction

Beaker 2: pure deionized water

Beaker 3: pure NMP

Beaker 4: pure NMP

Beaker 5: 1/2 NMP fraction, 1/2 deionized water fraction

1. Treat the S1818 structure on glass with oxygen plasma for 15 seconds at 80W.
2. Incubate the S1818 structure with 100μg/ml PLL-g-PEG in PBS solution for 1 hour at room temperature. Place the droplet of PLL-g-PEG solution on top of the coverslip, do not use a method with inverting the slide on parafilm as this decreases transfer of the PLL-g-PEG to the glass.

1. Wash each sample three times with 1 ml of deionized water. Place each coverslip in a carrier for batch processing. Make sure you are aware of which side has the photoresist (it is best to face the coverslips all the same way with some kind of reference on the coverslip holder).
2. Begin the lift-off procedure by dipping the carrier of coverslips into beaker 1 for 20 seconds; agitate the coverslip carrier with tweezers.

1. Transfer the coverslip carrier into beaker 2 and leave for 10 seconds.

1. Transfer the coverslip carrier immediately into beaker 3 and sonicate for 1 minute.

1. Transfer the coverslip carrier into beaker 4 and sonicate for 5 minutes.

1. Transfer the coverslip carrier into beaker 5 and sonicate for 1 minute.

1. Transfer the coverslip carrier into a 200 ml beaker filled with distilled water. Agitate with a magnetic stir bar for 5 minutes.
2. Take each coverslip out of the carrier one by one and rinse three times with deionized water. Make sure you know which side of the coverslip was patterned. Dab the edge of each coverslip on a kimwipe to eliminate extra water. Avoid drying the coverslip.
3. Incubate each coverslip with 100 μg/ml protein solution for 1 hour at room temperature.
4. Wash sample three times with PBS and leave in PBS until use.

**Microcontact printing of glass coverslips**

1. Create a mold using SU8 2010 photoresist on a silicon wafer. First, spin a uniform base layer (10 µm thickness) to increase adhesion of photoresist to the wafer and then use photolithography and a chrome mask (again with SU8 2010) to create the desired features. Follow Microchem manufacturer’s suggestions for spinning, exposure dose, and baking parameters to get a 9 µm height feature layer.
2. Fabricate polydimethylsiloxane (PDMS) ‘stamps’ of features from a photoresist mold on a silicon wafer. Use 10:1 formulation of Sylgard 184.
3. Dice the PDMS wafer into 1 x 1 cm PDMS stamps containing the features of interest. Clean the PDMS stamps with an air gun and pipette a 100 µg/ml protein solution on top of the stamps. Spread the solution out to cover the stamp using a micropipette tip. Incubate the solution for an hour.
4. Aspirate the protein solution off the stamps with a pipette tip on vacuum tubing. Draw liquid off all of the sides without touching the patterned region. Use low nitrogen air flow to dry the stamps until all condensation disappears.
5. Use glass coverslips which have been cleaned overnight in 2% Hellmanex. Place the coverslip on a clean surface and then gently place your stamp straight down onto the coverslip. Avoid smearing and re-centering if you already touched the PDMS to the glass surface. Lightly tap the stamp with tweezers to initiate contact spreading. Keep the coverslip and PDMS together for 5 minutes.
6. Using tweezers, firmly grasp the edge of the coverslip and lift it off the stamp surface (avoid smearing and loosing traction on the coverslip – proceed slowly). Place coverslip in a Petri dish with the patterned side up.

**Polyacrylamide Gels Preparation on glass substrates**

1. Prepare functionalized coverslips to bind polyacrylamide by mixing the bind-silane working solution (3 µl Bind-Silane, 50 µl acetic acid, and 50 µl 95% ethanol) and applying it to coverslips for 5 minutes. Then rinse the coverslips with ethanol and allow to dry in the dessicator.
2. Follow S1 Table to determine the amount of acrylamide and bis-acrylamide (crosslinker) needed to create the desired stiffness gel. Pipette the water, acrylamide, and bis-acrylamide solutions into a centrifuge tube and take care not to introduce air bubbles while pipetting. Weigh out ammonium persulfate (APS) powder and create a 10% solution (w/v) using milliQ water.
3. Degas the mixture in the desiccator for 1 hour.
4. After degassing, take one tube of gel precursor at a time and add 5 µl of APS solution and 1 µl of TEMED (in that order). Mix well using the pipette tip but do not introduce bubbles.
5. Pipette the gel precursor mix onto the silanized coverslip and sandwich with protein patterned coverslip using 23 µl of solution for 18 mm round coverslips to yield a polyacrylamide gel 80 µm in height.
6. Wait for 30 min for polymerization at room temperature.
7. Following polymerization, immerse the gels in PBS overnight. Lift up the coverslip in PBS with a sharp tweezer when ready to use the gel surface for cell seeding or imaging of protein transfer. Store the gels in PBS in 4°C.

**Comparisons between polyacrylamide pore size and gelatin protein molecular size**

The volume of a gelatin molecule is smaller than the pore mesh size of all of the gel formulations used in our study and thus the molecule can diffuse into the matrix for any of the gels we used. Thus, when we visualize the gelatin using a fluorescent marker which is conjugated to it (Oregon Green), it must be attached to the gel as otherwise it would be free to diffuse in the surrounding medium and gel material.

Using relations by Holmes and Stellwagen [1] for polyacrylamide materials used for electrophoretic molecular sieving [2], we note that our 25 kPa, 10 kPa, and 5 kPa gel formulations correspond to effective mean gel pore radius of 70 - 103 nm, 112 nm, and 100 nm, respectfully. These calculations were verified by measurements by Wen [3] and Hsu [4] of pore sizes ranging from 88 – 166 nm for similar gel formulations. Following methods outlined by Erikson [5], we calculate each gelatin protein molecule (when modeled as a globular protein of 100 kDa) to have a volume of 121 nm^3^, a minimum radius of 3.06 nm, and a Stokes radius of 12.57 nm given a diffusion coefficient of 1.75E-7 cm^2^/s [6].

**References**

1. Holmes DL, Stellwagen NC. Estimation of polyacrylamide gel pore size from Ferguson plots of linear DNA fragments. II. Comparison of gels with different crosslinker concentrations, added agarose and added linear polyacrylamide. Electrophoresis. 1991;12(9):612-9. doi: 10.1002/elps.1150120903. PubMed PMID: 1752240.

2. Stellwagen NC. DNA mobility anomalies are determined primarily by polyacrylamide gel concentration, not gel pore size. Electrophoresis. 1997;18(1):34-44. doi: 10.1002/elps.1150180108. PubMed PMID: 9059818.

3. Wen JH, Vincent LG, Fuhrmann A, Choi YS, Hribar KC, Taylor-Weiner H, et al. Interplay of matrix stiffness and protein tethering in stem cell differentiation. Nat Mater. 2014;13(10):979-87. doi: 10.1038/nmat4051. PubMed PMID: 25108614; PubMed Central PMCID: PMCPMC4172528.

4. Hsu T-P, Cohen C. Observations on the structure of a polyacrylamide gel from electron micrographs. Polymer. 1984;25(10):1419-23. doi: 10.1016/0032-3861(84)90103-4. PubMed PMID: 6943988613727681983related:v32YddoAXmAJ.

5. Erickson HP. Size and shape of protein molecules at the nanometer level determined by sedimentation, gel filtration, and electron microscopy. Biol Proced Online. 2009;11:32-51. doi: 10.1007/s12575-009-9008-x. PubMed PMID: 19495910; PubMed Central PMCID: PMCPMC3055910.

6. Yoshimura K, Terashima M, Hozan D, Ebato T, Nomura Y, Ishii Y, et al. Physical properties of shark gelatin compared with pig gelatin. J Agric Food Chem. 2000;48(6):2023-7. PubMed PMID: 10888492.
