# Supplementary figures and images for: Controlling cell shape on hydrogels using lift-off protein patterning
Source: PLoS One. 2018 Jan 3;13(1):e0189901. doi: 10.1371/journal.pone.0189901 (PMC5752030; doi:10.1371/journal.pone.0189901)

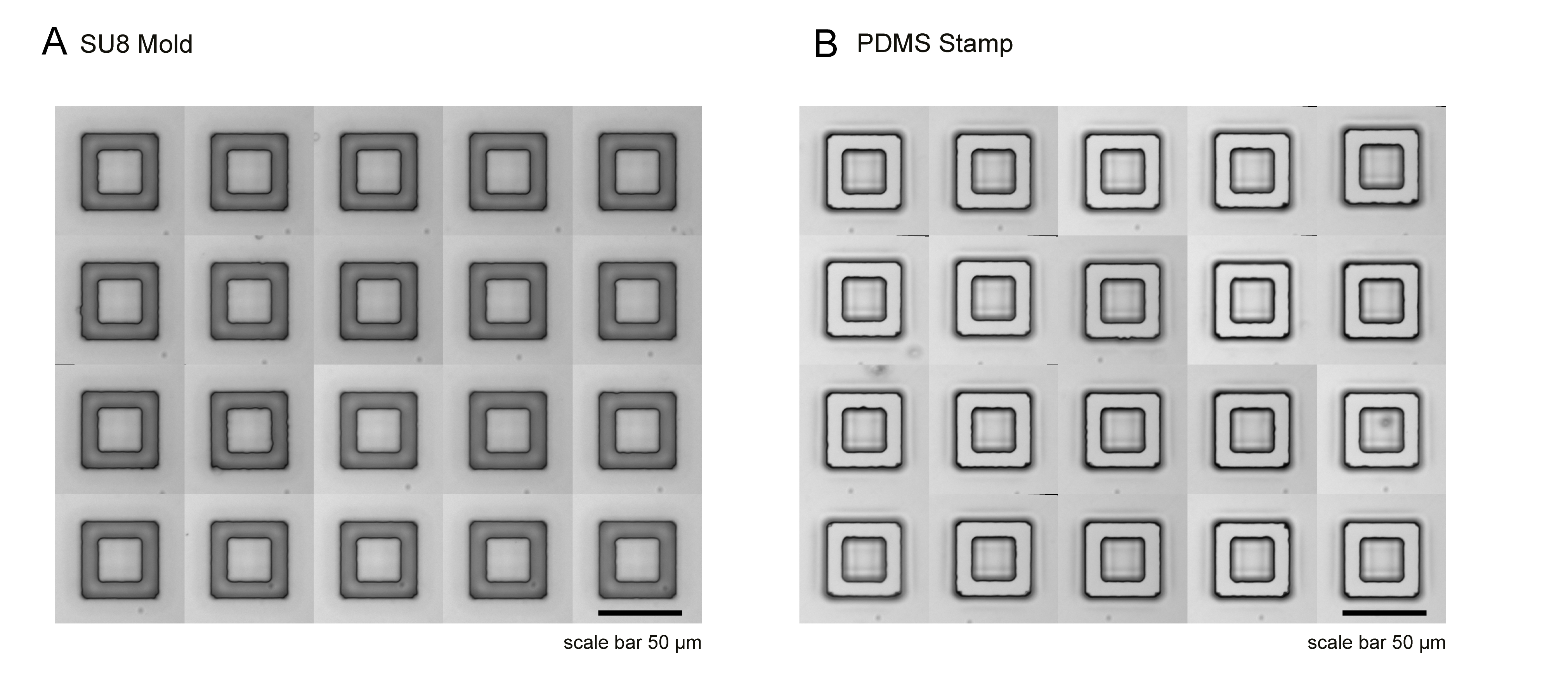

Supplement: S1 Fig — The photolithography mold (A) and PDMS stamp cast from this mold (B) show rounded corners where the edges of the pattern meet, both in the inner and outer regions of the pattern. The height of the SU8 mold (~9 μm) may be limiting the pattern accuracy achievable with microcontact printing. (TIF) [file pone.0189901.s004.tif]

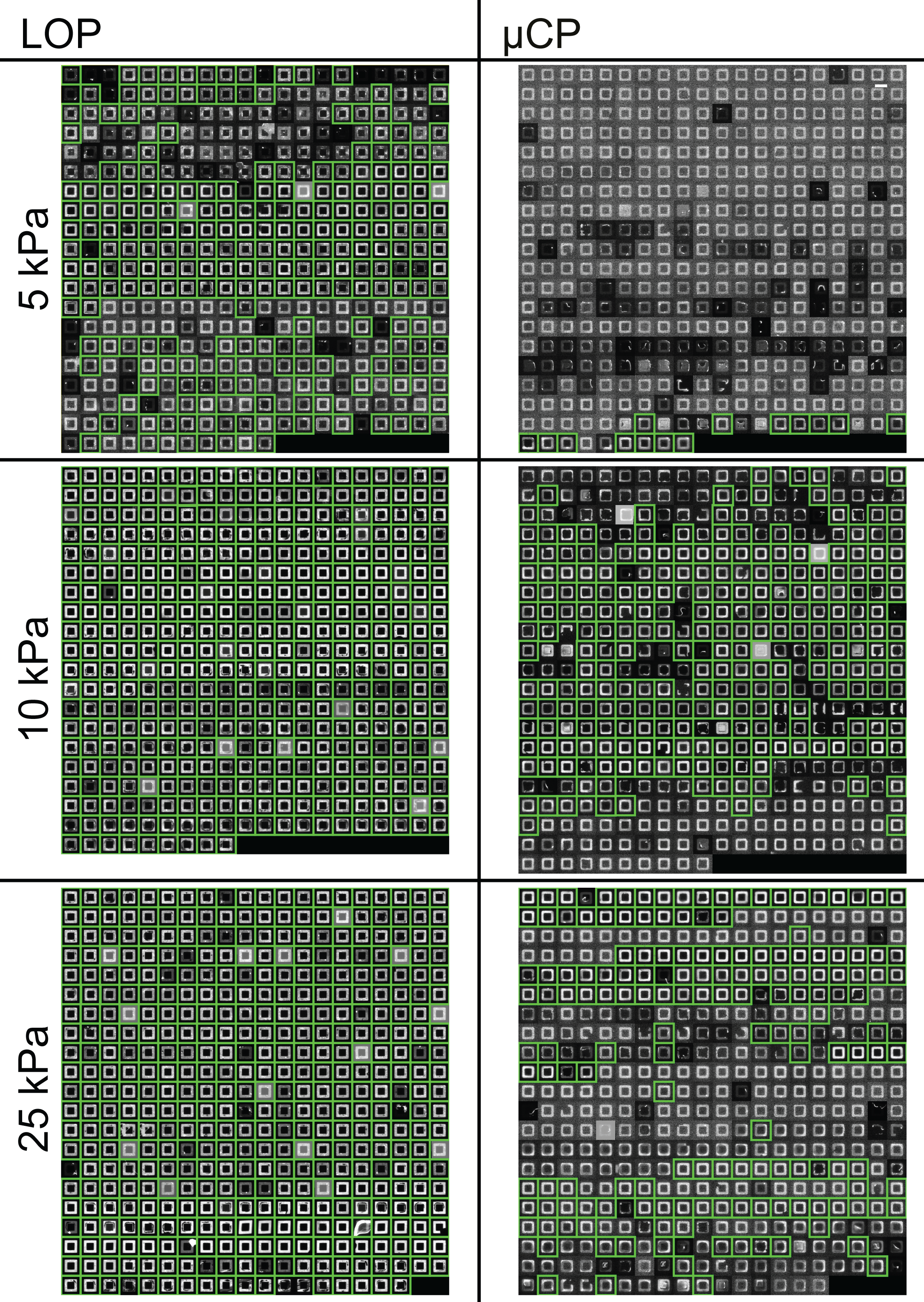

Supplement: S2 Fig — By setting a threshold of a 0.84 correlation coefficient, the LOP protocol resulted in more acceptable features than μCP (highlighted in green). We selected 389–416 features for each gel sample and then performed cross correlation analysis on the collected feature montage. Acceptable feature yield varied from 59% to 98% for LOP and from 4% to 72% for μCP for different gel formulations. See S2 Table for summary of data. (TIF) [file pone.0189901.s005.tif]

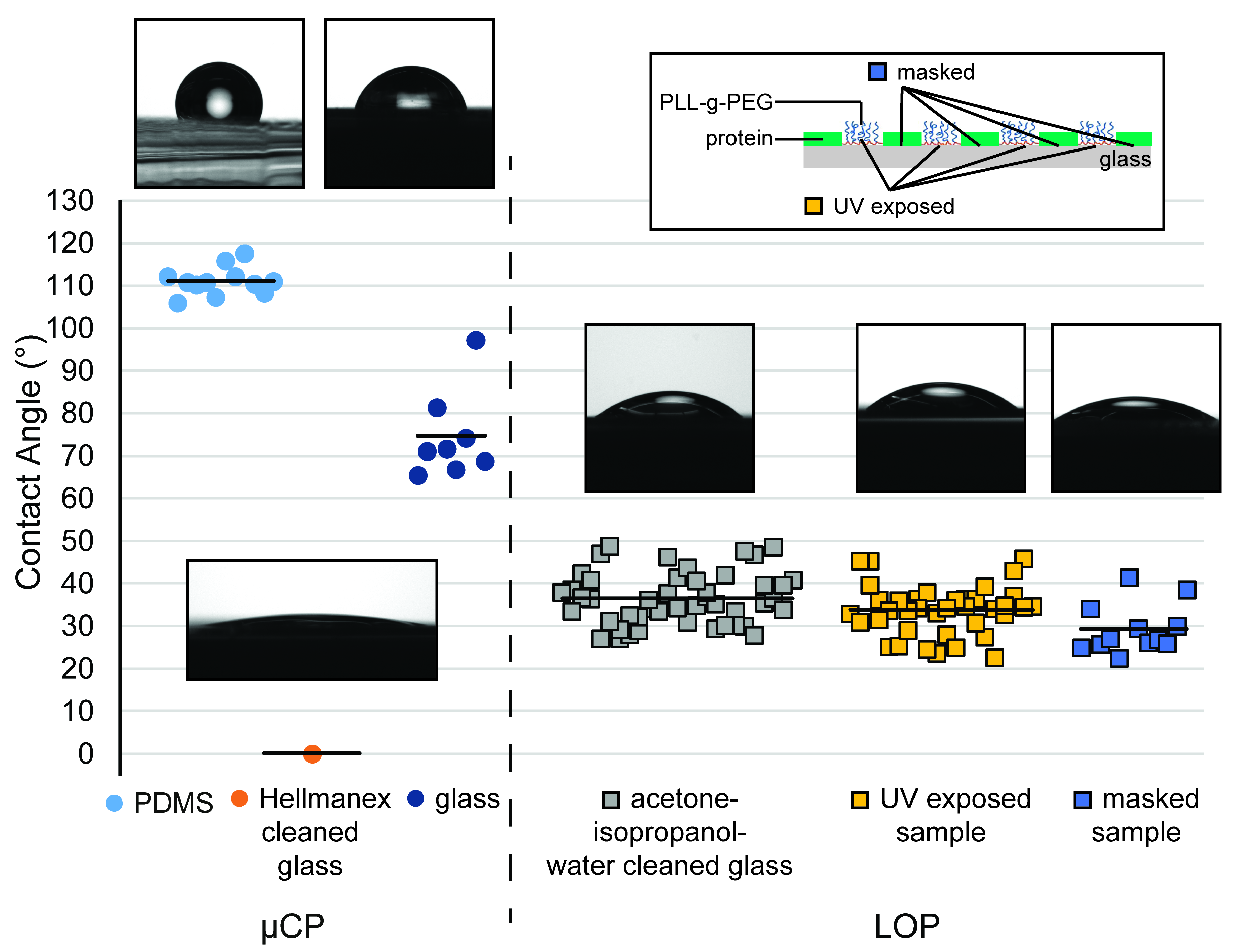

Supplement: S3 Fig — The water contact angle of substrates used in μCP differs substantially from average of 111° for PDMS (n = 12 measurements) to approximately 0° for Hellmanex-cleaned glass (the substrate used for μCP). The Hellmanex treated glass sample was super hydrophilic making an exact measurement of the low water contact angle difficult. Untreated glass is shown as comparison with an average water contact angle 75° (n = 8 measurements). The substrates used for LOP varied little in water contact angle. The “UV-exposed” sample corresponds to glass cleaned with acetone-isopropanol-water, coated with S1818 resist, flood-exposed to UV, developed, and processed with NMP for lift-off. In the LOP protocol, areas that adsorb the PLL-g-PEG adlayer have been treated with the same procedure. The “masked” sample corresponds to glass cleaned with acetone-isopropanol-water, coated with S1818 resist, no UV exposure, developed, and processed with NMP for lift-off. This substrate thus replicates the surface areas that adsorb protein in the LOP protocol. See insets from our LOP protocol and mask design for clarification. We recorded average water contact angles of 36° for glass cleaned in a series of acetone-isopropanol-water (n = 48 measurements), 34° for “UV exposed” samples (n = 38 measurements), and 29° for “masked” samples (n = 12 measurements). For μCP, protein must be transferred from the hydrophobic PDMS to the hydrophilic Hellmanex-cleaned glass. For LOP, protein would be adsorbed to the areas masked by S1818 after those areas are exposed by lift-off and we found these areas to be hydrophilic. Insets show examples of water droplets on the corresponding substrates. (TIF) [file pone.0189901.s006.tif]

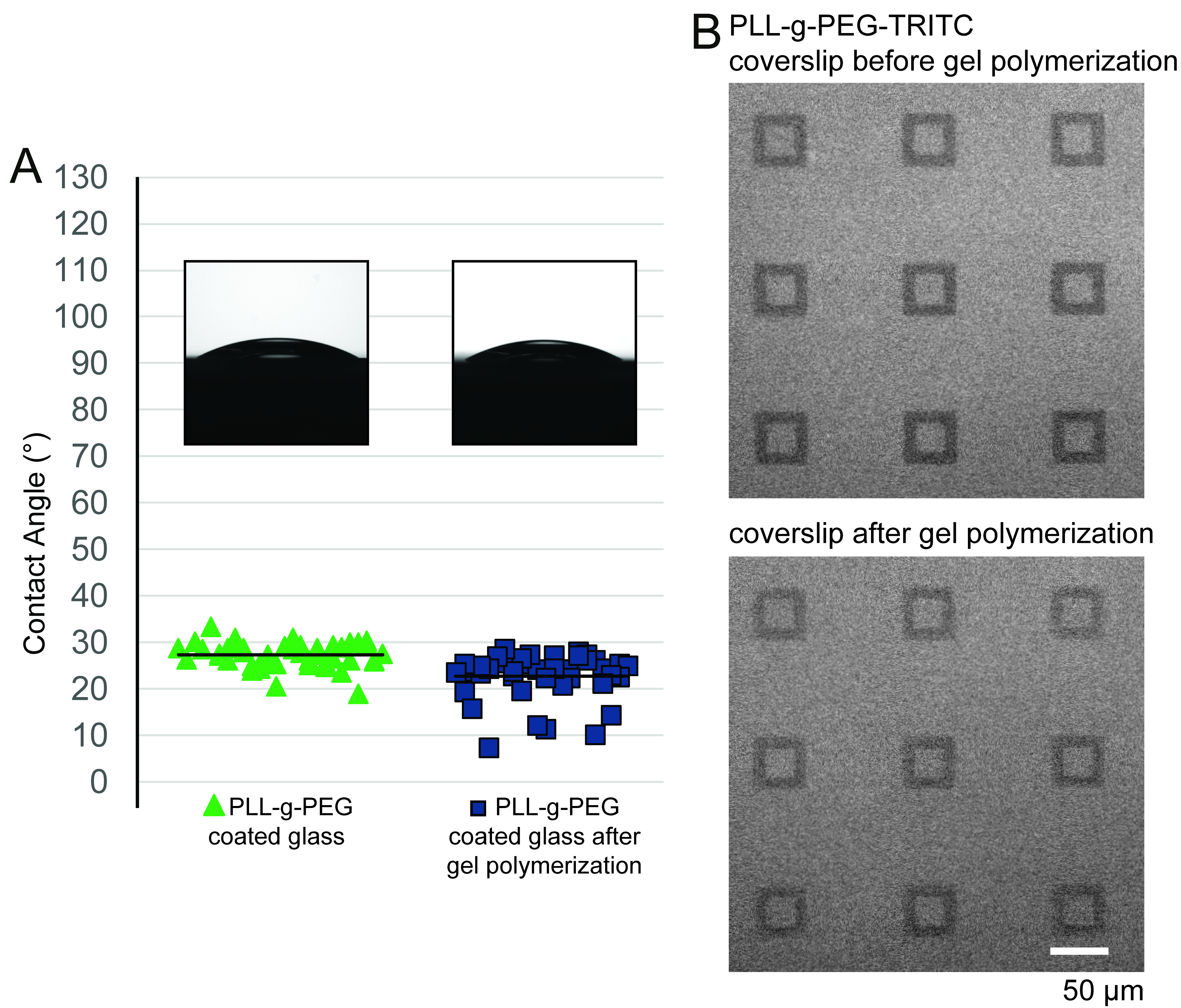

Supplement: S4 Fig — A.) We measured the contact angle of PLL-g-PEG coated glass before and after polymerizing a polyacrylamide gel. The average water contact angle is similar with 27° for PLL-g-PEG glass (n = 46 measurements) and 23° for PLL-g-PEG glass after gel polymerization (n = 42 measurements). B.) We also used TRITC-labeled PLL-g-PEG on the LOP patterned glass and measured the intensity of the fluorescent signal before and after gel polymerization on the same coverslip. We show a representative image showing the PLL-g-PEG-TRITC signal outside of the protein features (dark frames in image). We subtracted the signal within the protein pattern areas and divided the average PLL-g-PEG-TRITC signal ‘after’ gel polymerization by the ‘before’ signal. Within the limits of the measurement, no loss in PLL-g-PEG-TRITC intensity on the glass coverslip was observed (average 98% ± 2.6% of the initial signal remains on the glass after gel polymerization, n = 80 regions analyzed). We were also unable to detect PLL-g-PEG on the surface of the resulting polyacrylamide gels. Together, our water contact angle and fluorescence imaging data strongly suggest that PLL-g-PEG is not transferred to the PAAm gel during LOP. (TIF) [file pone.0189901.s007.tif]
